# Genome-wide association study unravels the genetic control of the apple volatilome and its interplay with fruit texture

**DOI:** 10.1093/jxb/erx018

**Published:** 2017-02-24

**Authors:** Brian Farneti, Mario Di Guardo, Iuliia Khomenko, Luca Cappellin, Franco Biasioli, Riccardo Velasco, Fabrizio Costa

**Affiliations:** 1 Research and Innovation Centre, Fondazione Edmund Mach, via Mach 1, 38010 San Michele all’Adige, Trento,Italy; 2 Graduate School Experimental Plant Sciences, Wageningen University, PO Box 386, 6700 AJ Wageningen, The Netherlands; 3 Institute for Ion Physics and Applied Physics, University of Innsbruck, Technikerstr. 25/3, 6020 Innsbruck, Austria

**Keywords:** Ester, functional principal component analysis, fruit texture, GWAS, multiple factor analysis, PTR-ToF-MS, phenylpropene, SNP, volatilome, VOCs.

## Abstract

Fruit quality represents a fundamental factor guiding consumers’ preferences. Among apple quality traits, volatile organic compounds and texture features play a major role. Proton Transfer Reaction-Time of Flight-Mass Spectrometry (PTR-ToF-MS), coupled with an artificial chewing device, was used to profile the entire apple volatilome of 162 apple accessions, while the fruit texture was dissected with a TAXT-AED texture analyzer. The array of volatile compounds was classed into seven major groups and used in a genome-wide association analysis carried out with 9142 single nucleotide polymorphisms (SNPs). Marker–trait associations were identified on seven chromosomes co-locating with important candidate genes for aroma, such as *MdAAT1* and *MdIGS*. The integration of volatilome and fruit texture data conducted with a multiple factor analysis unraveled contrasting behavior, underlying opposite regulation of the two fruit quality aspects. The association analysis using the first two principal components identified two QTLs located on chromosomes 10 and 2, respectively. The distinction of the apple accessions on the basis of the allelic configuration of two functional markers, MdPG1 and MdACO1, shed light on the type of interplay existing between fruit texture and the production of volatile organic compounds.

## Highlight

VOC production and fruit texture, assessed by multiple factor analysis, showed a contrasting behavior in apple. A GWAS approach dissected regulation of the volatilome and identified QTLs co-locating with important candidate genes.

## Introduction

Fruits are important components of the human diet, supplying important elements such as sugars, organic acids, vitamins and fiber. These elements change during the fruit developmental process to render the fruit more attractive and palatable, especially at the onset of ripening. Overall, the array of these compounds defines the quality of a fruit, as a degree of excellence (Klee, 2010). Fruit quality can therefore be defined by four principal quality factors: appearance, flavor, texture and nutritional properties (Costa *et al.*, 2011). Among these, appearance, texture and flavor directly impact the post-harvest performance and consumers’ appreciation (Harker *et al.*, 2008; Cliff *et al.*, 2016) and therefore the marketability of the fruit.

Physiologically, fruit texture depends on the dismantling process occurring on the polysaccharide cell wall architecture co-ordinated by cell wall-modifying proteins. Fruit texture is composed of a series of subtrait components, classified into mechanical and acoustic properties (Varela *et al.*, 2006; Costa *et al.*, 2011, 2012). Beside the preference of consumers for crisp and juicy apples, firmer fruit are also more amenable to long-term storage, facilitating shipping and ensuring a timely availability in the fruit market. In addition, fruit flavor is also another important quality factor, which results from the combination of a large array of primary and secondary metabolites. While taste is primarily related to non-volatile metabolites (mainly sugars, organic acids, free amino acids, and salts), aroma is represented by the interaction of a blend of volatile organic compounds (VOCs) with human receptors. The apple aroma depends on the interaction of >370 VOCs (Dimick and Hoskin, 1983; Fuhrmann and Grosch, 2002; Ulrich and Dunemann, 2012; Farneti *et al.*, 2015*a*) synthesized by the fruit during ripening and enhanced upon cellular disruption by biting and mastication (Contreras and Beaudry, 2013; Farneti *et al.*, 2015*b*). Among them, only a minor set of chemical compounds, mostly esters, alcohols, and aldehydes, can be distinctly perceived (Holland *et al.*, 2005; Ulrich and Dunemann, 2012).

Although these characteristics are essential factors for excellent fruit quality, breeding efforts have historically been mainly oriented towards improving fruit appearance and storability. Selection for yield, fruit size, color, and shelf-life properties might have had unintended negative consequences on other fruit quality traits, for instance aroma, as already suggested for strawberry, peach, and tomato (Goff and Klee, 2006; Klee, 2010; Rambla *et al.*, 2014). The selection of firm apple accessions, distinguished by a higher storability, has been also facilitated by the identification and subsequent validation of quantitative trait loci (QTLs) and functional markers, associated with both ethylene and texture (Costa *et al.*, 2005, 2010; Zhu and Barritt, 2008; Longhi *et al.*, 2012, 2013; Baumgartner *et al.*, 2016). Moreover, this drop in quality has been exacerbated by the fact that breeding for aroma occurred practically by chance (not assisted), since aroma is not considered as a discriminating trait in the early selection phase. This situation is also strengthened by the complex and time-consuming phenotyping protocols ordinarily used, which makes the analytical screening of large plant material unfeasible. This limitation reduced the number of scientific reports on QTL mapping related to apple aroma (Dunemann *et al.*, 2009, 2012; Rowan *et al.*, 2009; Kumar *et al.*, 2015; Yauk *et al.*, 2015). In most cases, VOCs have been monitored and quantified with solid-phase microextraction (SPME)-GC-MS equipment. Although this represents a valuable and accurate technique, it is laborious and time consuming. Therefore, Proton Transfer Reaction-Time Of Flight-Mass Spectrometry (PTR-ToF-MS) might represent a valid alternative to profile VOCs in a more time-efficient way (Lindinger *et al.*, 1998; Jordan *et al.*, 2009) also in apple (Zini *et al.*, 2005; Costa *et al.*, 2013; Cappellin *et al.*, 2015). Beside the headspace concentration of VOCs, the interaction between aromatic compounds and human receptors should also be considered (Farneti *et al.*, 2015*b*). Differences in VOC-releasing behaviors, due to the textural and physicochemical properties of the food matrix, may influence the perception of aroma during food consumption (Farneti *et al.*, 2013). VOCs are in fact released from the food matrix and then transported to receptors in the mouth and nose (Buettner *et al.*, 2008). The modification of the food matrix and the long incubation time normally required by static-based methodologies can drastically alter the *in vitro* VOC profile (Dewulf *et al.*, 2002; Biasioli *et al.*, 2011; Farneti *et al.*, 2013). The employment of a strategy suitable to monitor the VOC emission released during chewing is therefore preferable. To this end, Farneti *et al.* (2013, 2015*a*) developed an analytical system based on an artificial chewing device coupled to a PTR-Mass spectrometer in order to detect the VOC kinetics during food matrix processing.

In this survey, an apple collection was employed and assessed for both texture and aroma. To date, these two fruit quality traits have only been assessed separately, thus a comprehensive and exhaustive investigation of their relationship is lacking. The analysis of these data sets using multivariate statistical approaches [multiple factor analysis (MFA) and functional principal component analysis (FPCA)], together with a high-density single nucleotide polymorphism (SNP) genotyping platform, enabled a genome-wide association study (GWAS) with the aim of making progress in understanding the genetic relationship between these two important fruit quality traits.

## Materials and methods

### Plant material

In this investigation, a collection of 162 apple accessions was chosen within the germplasm repository available at the Fondazione Edmund Mach (Trento, Italy). Each genotype, planted in triplicate, was in the adult and fruit-bearing phase at the time of the analysis. Trees were maintained with standard agronomic practices for fruit thinning, pruning, and pest/disease control. Apples were harvested at the commercial ripening stage determined following the changes of skin and seed color as well as the degradation of the chlorophyll content assessed non-destructively with a Da-Meter (TR, Forli, Italy; Ziosi *et al.*, 2008). For each apple accession, a minimum of 20 homogeneous fruit were collected and stored for 2 months in a cold cellar (2–4 °C with ~95% relative humidity). After post-harvest storage, fruit were removed and maintained at room temperature (~20 °C) overnight before texture and VOC analysis.

### Fruit texture phenotyping

A subset of five apples per genotype was used for the characterization of the fruit texture subtraits. The measurements were carried out with a Texture Analyzer (Stable MicroSystem, Godalming, UK). The protocol was given in detail in Costa *et al.* (2011, 2012). Briefly, for each genotype, 20 measurements (four technical per five biological replicates) were carried out. The instrument was equipped with a 4 mm flat head probe and an AED (Acoustic Envelope Device) for the simultaneous acquisition of the mechanical and acoustic profiles, further processed with an ad hoc macro for the digital definition of 12 parameters. Out of these, eight were related to the mechanical signature of texture (yield force, maximum force, final force, mean force, area, force linear distance, Young’s modulus, and number of force peaks) and four to the acoustic response (maxim acoustic pressure, mean acoustic pressure, acoustic linear distance, and number of acoustic peaks; fully described in Costa *et al.*, 2011 and in Supplementary Table S1 at *JXB* online).

### Dynamic VOC fingerprinting in apple

**Fig. 1.**
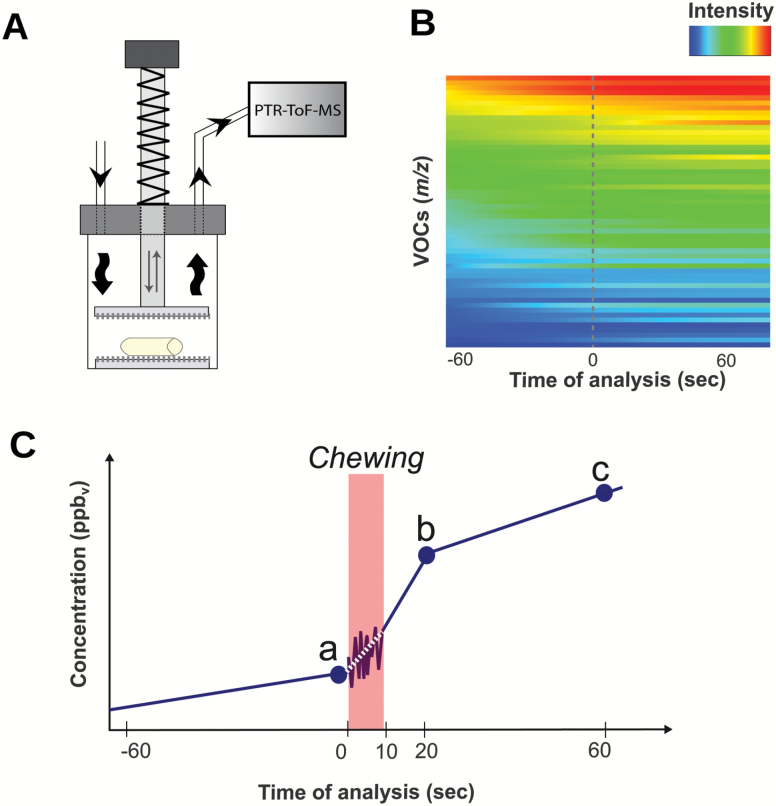
Schematic representation of the dynamic VOC fingerprinting of apple fruit assessed by PTR-ToF-MS coupled with an artificial chewing device (A) composed of a cylindrical glass cuvette (800 ml) sealed with a cap and a notched plunger. In (B) the 3D heatmap of the online VOC dynamic fingerprinting carried out in ‘Golden Delicious’, selected as reference cultivar, is reported. For graphic purposes the VOCs are reported in decreasing ordered based on their initial level (before chewing). The headspace VOC concentration was measured for 60 s before and after crushing, for a total of 120 s. In order to simplify the analysis of the entire dynamic VOC profiling, only three specific time points of the entire dynamics were primarily compared: (a) before the artificial chewing (0 s), (b) 20 s, and (c) 60 s after the fruit processing.

To profile the emission of VOCs during artificial chewing, another batch of five apples per genotype was assessed according to the method described by Farneti *et al.* (2015*b*). The chewing device was composed of a cylindrical glass cuvette (800 ml) sealed with a cap and equipped with a manual notched plunger (Fig. 1A). All device elements were made of polytetrafluoroethylene. The fruit sample was represented by a cylinder of apple flesh (1.7 cm diameter and 5 cm height) isolated from each fruit. Before crushing, the headspace VOC concentration of the apple flesh cylinder was measured for 60 s. The artificial chewing was performed by pressing the notched plunger five times within 10 s, and VOC analysis continued for 60 s following crushing (Fig. 1B, C). This setting was optimized in preliminary trials in order to ensure a variability <5% for analysis repeated on the same fruit. The headspace content was drawn from the chewing device to the PTR-ToF-MS at 2.4 l h^–1^. VOCs were then assessed by direct injection of the headspace mixture into a commercial PTR-ToF-MS 8000 apparatus (Ionicon Analytik GmbH, Innsbruck, Austria), set with the conditions described in Farneti *et al.* (2015*b*). All apple cultivars were measured in five independent replicates for each measurement data point. The analysis of PTR-ToF-MS spectral data, compound annotation, spectra correction through Poisson statistics, internal calibration, noise reduction, baseline removal, and compound quantification proceeded according to Cappellin *et al.* (2011*a*, b, 2012).

### SNP genotyping

Young leaves collected from each apple accession were used for nucleic acid extraction. Genomic DNA was isolated with a Qiagen DNeasy Plant Kit. DNA quantity and quality were measured with a Nanodrop ND-8000 (ThermoScientific, USA). The 162 accessions were genotyped with the 20K Infinium SNP Array (Illumina), ad hoc designed for apple (Bianco *et al.*, 2014). SNP data were filtered with ASSIsT (Di Guardo *et al.*, 2015) obtaining a final set of 11 277 polymorphic markers. However, the identification and selection of the final set of markers to be used in the GWAS cannot rely exclusively on SNP qualitative parameters (e.g. relative amount of missing calls, call rate ratio, and segregation distortion), since the physical position of a marker may turn out to be inaccurate, due to the high sequence homology between homoeolog chromosomes and the high heterozygosity of the apple genome (Velasco *et al.*, 2010). Thus, from the total number of SNP markers included in the 20K Illumina Infinium Array, 9142 were finally employed for GWAS analysis. This subset was therefore reliable within the 162 apple accessions (based on the filtering processed by ASSIsT) and positioned on the consensus genetic map described in the accompanying manuscript (Di Guardo *et al.*, 2017).

### Marker–trait association by GWAS

Filtered SNP data and phenotypic assessment (represented by both texture and VOC analysis) were jointly analyzed in a marker–trait association approach. For this purpose, the software TASSEL v3.0 was employed and the GWAS was computed implementing two models: the general linear model (GLM) and the mixed linear model (MLM). The GLM (Pritchard *et al.*, 2000) was performed taking into consideration population structure (Q matrix) to correct for genetic stratification. The membership of each individual in each subpopulation, represented by principal components (PCs), was further added to the model as covariates. The second model adopted here to find marker–trait associations was the MLM (Yu *et al.*, 2006), which also considered the Kinship matrix (population relatedness) to correct for false association. This model is expressed by the Hendersen matrix as follows:

Y=Xβ+Zu+e

where *Y* is the vector of observation, β is a vector containing fixed effects (including genetic markers and population structure), *u* is a vector of random additive genetic effects for multiple background QTLs, *X* and *Z* are the known design matrices, and *e* is the unobserved vector of random residuals. Significant associations were selected on the threshold of *P*-value ≤0.05, corrected for multiple comparisons according to the false discovery rate (FDR) procedure reported by Benjiamini and Hochberg (1995), calculated with the ‘stats’ package of R (R Core Development Team). The model used for each trait was selected on the basis of the visual inspection of the Q–Q plot (‘qqman’ R package).

### Statistical analysis

The array of protonized VOC masses was reduced by applying noise and correlation coefficient thresholds. The first removed peaks with mean intensity <25 ppbv and not significantly different from blank samples (Farneti *et al.*, 2015*a*). The latter excluded peaks having correlation >99%, which corresponds mostly to isotopes of monoisotopic masses. During the progression of the notched plunger, the VOC profile/signal was, as expected, not stable; thus, 10 s of artificial mastication were removed and substituted by cubic spline interpolation (Fig. 1C). Further analyses were therefore carried out with smoothed curves. Each VOC dynamic was characterized by the mean intensity at three specific time points: before chewing (a, 55 ± 5 s), immediately after chewing (b, 80 ± 5 s), and at the end of the measurement (c, 120 ± 5 s). To represent the general changes of VOC profiles, principal component analysis (PCA) was performed on the log-transformed data on these three data points. An MFA was used to compare fruit texture further with VOC profiles before fruit crushing, in a way similar to classical static headspace analysis. Moreover, a multivariate functional principal component analysis (FPCA; Ramsay and Silverman, 2005) was used to perform the analysis on the whole VOC pattern released during the artificial chewing. To this end, 50 linear combinations of parabolic b-spline basis objects (the highest order equal to 3) were constructed creating new curves defined as functional data objects from the VOC data set.

Visualization of significant VOC correlations (*P*<0.01; *R*>0.50) was conducted by the generation of a correlation analysis network with Cytoscape (version 3.2.1; Cline *et al.*, 2007). The ClusterONE plugin (Nepusz *et al.*, 2012) was used to identify putative metabolite clusters by finding regions of significant local density. R 3.2.0 internal statistical functions and the external packages ‘ChemometricsWithR’, ‘FactoMineR’, ‘Funclustering’, ‘fda’, and ‘ggplot2’ were used for the multivariate statistical methods employed in this work. Regarding the texture analysis, each combined mechanical–acoustic profile was analyzed with the Exponent v4.0 software (Stable MicroSystem, Godalming, UK).

## Results and Discussion

### High-resolution VOC phenotyping

In this study the interplay between fruit texture and aroma was investigated by a comprehensive high-resolution phenotyping assessment (Supplementary Table S2). Fruit were analyzed after a period of cold storage (2 months), also taking into account that both the volatilome and texture undergo important changes during the post-harvest phase (Fellman *et al.*, 2003; Newcomb *et al.*, 2006; Schaffer *et al.*, 2007). The analysis of aroma was performed on apple cut flesh portions, since the release of aroma is distinct and dominating in processed fruit (for instance, during mastication) rather than when intact (Farneti *et al.*, 2015*b*). It is in fact known that VOCs can indeed be distinguished in two categories (Yahia, 1994): those produced by whole fruit and those synthesized during chewing. Fruit cutting greatly stimulates the changes in both concentration and composition of VOCs in the headspace, due to an increased exposure of the food matrix to air (de Roos, 2003; Arvisenet *et al.*, 2008). This operation triggers several chemical reactions as a consequence of cell disruption, such as lipid oxidation and the consequent synthesis of aldehydes. In order to evaluate the VOCs affecting aroma perception, the methodology proposed by Farneti *et al.* (2015*b*), based on the real-time analysis of volatiles emitted during *in vitro* mastication, was used (Fig. 1A). The PTR-ToF-MS setting employed in this investigation enabled a full scan of the entire volatilome in 1 s, allowing real-time monitoring for most VOCs emitted by the fruit during chewing. This detailed characterization permitted the development of a dynamic VOC fingerprint before and after mastication of the fruit *in vitro* (Fig. 1B; Supplementary Fig. S1). The entire VOC profile, assessed for the 162 apple accessions by PTR-ToF-MS, was reduced from 590 to 33 masses, applying noise and correlation coefficient thresholds. The resulting array of VOCs (Supplementary Table S3) was comparable with the data set described by Farneti *et al.* (2015*a*). The blend of VOCs detected in apple fruit for the most part includes alcohols (i.e. *m/z* 33.033, 47.049, or 85.101), aldehydes (i.e. *m/*z 83.086, 99.081, or 101.097), phenylpropenes (i.e. *m/z* 134.072 and 149.097), and esters (i.e. *m/z* 61.027, 89.059, or 117.091). Among these classes, esters, of both straight and branched types, are to date recognized as the most relevant VOCs in apple aroma (Holland *et al.*, 2005; Ulrich and Dunemann, 2012). Similarly to the results presented by Farneti *et al.* (2013, 2015*b*), VOCs were released differently from the food matrix according to their chemical nature and, more probably, to the textural properties of the apple flesh (Supplementary Fig. S1). Overall, esters (i.e. *m/z* 61.028, 89.059, and 117.091) and alcohols (i.e. *m/z* 43.054, 57.069, and 71.086) were rapidly released after crushing the fruit structure, while for other molecules, such as acetaldehyde (*m/z* 45.033) and acetone (*m/z* 59.049), the emission was less influenced by disruption of the sample. In addition to this, several other compounds, mainly C6-aldehydes, such as hexanal (*m/z* 83.086 and 101.097) and hexenals (*m/z* 81.070 and 99.081), revealed a third trend characterized by a constant and linear production, delayed by ~20 s after the initial tissue disruption (Supplementary Fig. S1).

**Fig. 2.**
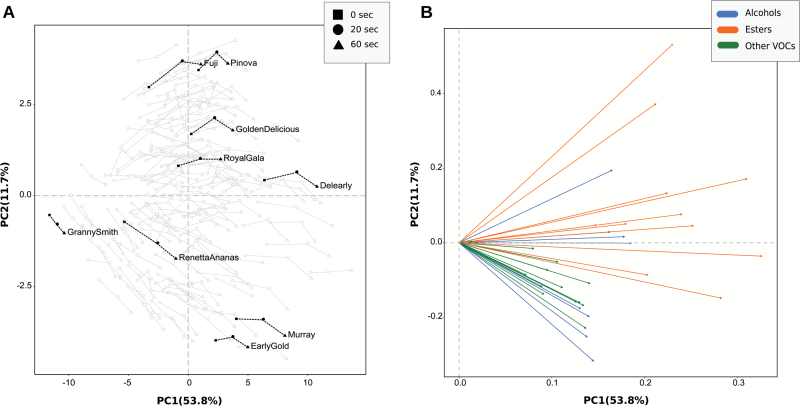
Principal component analysis (PCA) plot (A) and loading projection (B) of the VOC distribution assessed by PTR-ToF-MS during the artificial chewing. The plot in (A) depicts the VOC profile distribution of the apple cultivars over the PCA score plot defined by the first two principal components. Within the germplasm collection, nine cultivars were arbitrarily highlighted (‘Delearly’, ‘Early Gold’, ‘Fuji’, ‘Golden Delicious’, ‘Granny Smith’, ‘Murray’, ‘Pinova’, ‘Renetta Ananas’, and ‘Royal Gala’). Different symbols (square, circle, and triangle) indicate the time of assessment during the artificial chewing (0, 20, and 60 s after chewing). Each data point is the average of five biological replicates. The plot in (B) shows the projection of the 33 significant VOC mass peaks reported using different colors according to the chemical family. The mass peak identity is reported in Supplementary Fig. S2.

As a first attempt to simplify the analysis of the entire VOC profile during the *in vitro* mastication, we initially compared only three specific time points of the entire VOC dynamic: (i) before the artificial chewing (0 s); (ii) 20 s after the fruit processing; and (iii) 60 s after the fruit processing (Fig. 1C). The effect of the artificial mastication on apple VOC profiling is depicted in the PCA plot defined by the use of the first two principal components, together explaining 65.5% of the total apple volatilome variability (Fig. 2A; Supplementary Fig. S2). According to the loading plot (Fig. 2B), the first principal component (PC1, 53.8%) mainly correlates with the quantitative concentration of VOCs, while the second (PC2, 11.7%) was more related to the qualitative distinction of VOCs (chemical composition). Positive PC2 values indicate, for instance, a higher concentration of esters (i.e. *m/z* 61.027, 43.017, or 71.085) and amyl alcohols (i.e. *m/z* 71.085), while negative values are linked to a greater abundance of methanol (*m/z* 33.033), acetaldehyde (*m/z* 45.033), ethanol (*m/z* 47.049), and C6-aldehydes (*m/z* 81.07 or 83.086). Beyond the differentiation of apple cultivars based on their aromatic profiles, samples are also differentially distributed on the PCA-hyperspace according to the VOC assessment carried out at three specific time points after chewing (0, 20, and 60 s). Although the VOC magnitude was enhanced by mastication, a genetic and physical (apple flesh structure) regulation was suggested.

### Volatilome QTL mapping

**Fig. 3.**
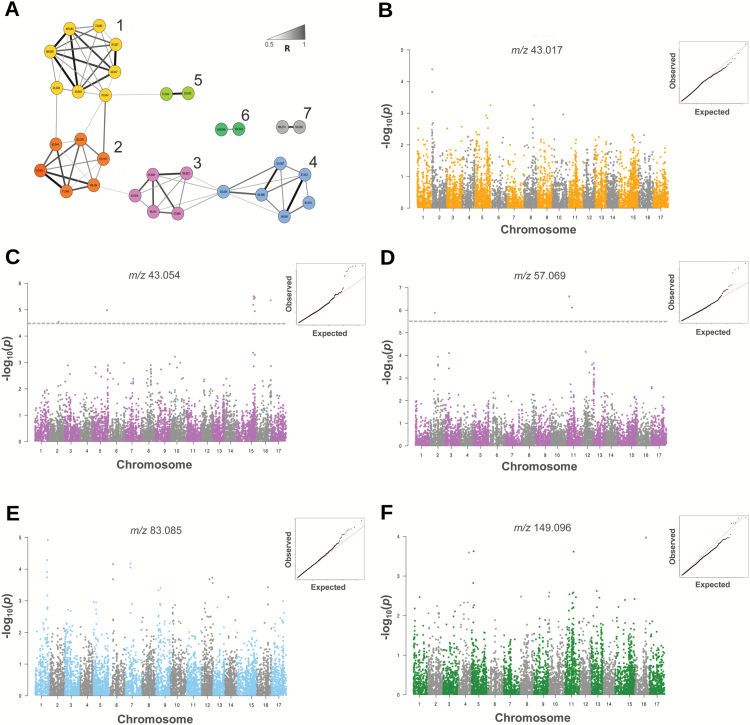
Correlation analysis networks (CANs) of VOCs (A) and genome-wide association results (from B to F). The CAN (A) is obtained by determining the significant Pearson correlations (*P*<0.01) among the 33 VOC mass peaks assessed by PTR-ToF-MS at 60 s after the artificial mastication. The color coding of the edges (gradient from light gray to black) denotes the level of correlation (*R* from 0.5 to 1). Positive and negative correlations are shown by solid and dashed lines, respectively. Significant VOC clusters, identified by Cytoscape ClusterONE plugin, are highlighted with different colors. Each cluster is also defined by a numerical code according to the VOC category as follows: 1, esters; 2, ethanol and acetaldehyde; 3, alcohols; 4, aldehyde; 5, methanol; 6, phenylpropenes; and 7, unknown compounds. For GWAS analysis, five Manhattan plots (from B to F) showing the SNP association (based on *P*-value –log_10_ transformed) with selected representative masses (reported on the top of each plot and depicted with the same color as the CAN grouping) are illustrated. For each Manhattan plot, the Q–Q plot and the corrected *P*-value threshold (FDR ≤0.05), when possible, are also indicated.

To reveal the general behavior existing among the VOCs assessed within the germplasm collection, the apple volatilome was analyzed through a correlation network (Fig. 3A). The network, created from a significant Pearson correlation matrix (*P*≤0.01, threshold 0.05) among the set of 33 masses, identified seven main groups of VOCs (Fig. 3A; Supplementary Table S3). The first two groups (1 and 2) mainly include ester compounds, ethanol, and acetaldehyde. The high positive correlation between esters and the two anaerobic metabolites (acetaldehyde and ethanol) is generally observed in several fruit species, since the latter compounds are involved in the synthesis of several aroma volatiles during fruit ripening (El Hadi *et al.*, 2013). Acetaldehyde is generally accumulated during ripening, also under aerobic conditions (Fidler, 1968), and it is formed from pyruvate by the action of the enzyme pyruvate decarboxylase (PDC). The two immediate products formed from acetaldehyde are ethanol, produced by alcohol dehydrogenase (ADH), and acetyl-CoA, obtained by the activity of the enzyme aldehyde dehydrogenase (ALDH; Cossins, 1978). While acetyl-CoA is the precursor of acetate esters, acyl-CoA is involved in the formation of longer esters (Gilliver and Nursten, 1976). The amount of ethyl esters (such as ethylacetate and ethyl butanoate) is correlated with the content of ethanol (Pesis, 2005). The third and fourth group of masses were mainly composed of alcohols and C6-aldehydes, respectively (Supplementary Table S3). The remaining three clusters (5, 6, and 7) were composed of methanol (*m/z* 33.033 and its water cluster *m/z* 51.044) and phenylpropenes (*m/z* 134.072 and 149.096), together with unknown fragments. Methanol, similarly to ethanol and acetaldehyde, is positively correlated (*R*=0.63) with an ester included in group 1, in agreement with the involvement of methanol in the methyl ester synthetic pathway (i.e. methyl acetate, *m/z* 75.044).

To identify the most significant genomic regions involved in the genetic control of the apple volatilome, a GWAS was performed with MLM. The genetic dissection was carried out by selecting the most reliable VOCs (mass) within each network analysis group, based on the Q–Q plot. The choice of the representative mass/cluster is also justified by the fact that the PTR-ToF-MS device accurately detects the nominal mass of a molecule, important to identify an array of compounds with similar structure and thus with a similar quality impact. The association between the set of SNPs and *m/z* 43.017, a common ester fragment selected to represent group 1, identified a major QTL on chromosome 2 (Fig. 3B; Supplementary Table S4). Although the most significant SNP (RB_1979331_L2_PA) does not cross the adjusted threshold, this genomic region coincided with *MdAAT1.* This gene belongs to the alcohol acyl-transferase family and it is known to catalyze the transacylation from acyl-CoA to alcohol (esterification). In several fruits, *AAT* is essential to control flavor biogenesis during the fruit ripening phase. It has in fact been documented (Aharoni *et al.*, 2000; Beekwilder *et al.*, 2004; El-Sharkawy *et al.*, 2005; Dunemann *et al.*, 2012) that esters are the most important compounds within the aromatic bouquet of fruit. In apple, moreover, esters are, amongst others, the dominating compound, contributing 80% of the entire aromatic blend. For this species, the expression profile of *MdAAT1* was shown to be consistent with the production of esters and also the accumulation of ethylene (Schaffer *et al.*, 2007). This gene (MDP0000637737) was further retrieved from the apple genome assembly (GDR database; Jung *et al.*, 2014) within an interval of 400 kb, established as the extent of linkage disequilibrium (LD) present in domesticated apples (Di Guardo *et al.*, 2017).

For the alcohol group (depicted in group 3; Fig. 3A), two masses were selected and further used in the marker–trait association study (*m/z* 43.054 and *m/z* 57.069). These two compounds were selected on the basis that they did not show a high correlation value (*R*=0.35), indicating the possibility of the identification of two distinct groups. For *m/z* 43.054, statistically significant SNPs were found in chromosomes 2, 5, 15, and 16 (Fig. 3C; Supplementary Table S4). Interesting candidate genes were identified in the homoeologous pair of chromosomes 2 and 15 (within the LD interval of 400 kb). In the QTL region on chromosome 2 an *alcohol dehydrogenase-1 like* (*ADH*) gene was annotated (MDP0000523942). The action of *A*DH is to reduce aldehydes (previously reduced by acyl-CoA) to alcohols (that will be further converted to esters by AAT; De Filippi *et al.*, 2005). In other climacteric species such as tomato, this gene is expressed during ripening (Chen and Chase, 1993), and functional validation demonstrated that fruit with an enhanced ADH activity were distinguished by a higher concentration of alcohols and a more typical flavor of ripe fruit (Speirs *et al.*, 1998). On the same chromosome region, *MdACS3* (MDP0000247533) was also identified. This element, which belongs to the *1-aminocyclopropane-1-carboxylate* family, is involved in the early phase of apple ethylene production (Wang *et al.*, 2009). Since its expression precedes *MdACS1* (mapped on chromosome 15; Costa *et al.*, 2005), this element might play an important role in the transition phase from ethylene system 1 to system 2, thereby supporting the direct role of this hormone in controlling the VOC production. On chromosome 15, instead, another *alcohol acethyl transferase* gene was identified (MDP0000528775), supporting the interplay between alcohols and esters in apple. Chromosomes 2 and 15 were already associated with the accumulation rate of alcohol compounds in apple by Kumar and colleagues (2015); however, no genes were identified in these regions. For the second alcohol compound, employed in the GWAS analysis (*m/z* 57.069), a QTL was located on chromosome 11 (Fig. 3D; Supplementary Table S4), on which a short-chain *dehydrogenase reductase3b-like* gene (*SDR*) was annotated (MDP0000313884). This gene encodes one NAD(P)(H)-dependent enzyme characterized by a wide range of substrates, including alcohols and aromatic compounds (Persson *et al.*, 1995; Kallberg *et al.*, 2002). This gene, also known as *alcohol dehydrogenase*, is involved in the regulation of the alcohol/aldehyde ratio (Moummou *et al.*, 2012). In particular, *SDR* genes contribute to biosynthesis of aroma compounds in tomato, converting phenyl acetaldehyde to the corresponding alcohol (Tieman *et al.*, 2012). SNP markers associated with these compounds (Supplementary Table S4) showed a *P*-value exceeding the statistical threshold corrected for multiple comparisons (FDR ≤0.05).

From the group of aldehyde compounds (group 4), QTLs were identified and located on chromosome 1 and 7, respectively (coincident with FB_0442970_L1_PA, FB_0697476_L7_PA; Fig. 3E; Supplementary Table S4). Although no relevant gene was identified for chromosome 1, an additional *ADH* gene (MDP0000077529) was annotated on chromosome 7. This association, together with the aforementioned reported genes, strengthens the role of this region in the regulation between alcohol and aldehyde VOC categories.

The last VOC implemented in the GWAS analysis is *m/z* 149.096 (Fig. 3F; Supplementary Table S4), corresponding to phenylpropenes. The ordering of the SNP markers based on their *P*-value allowed the detection of three genomic regions located on chromosome 5 (RB_14354679_L5_PA), 11 (FB_0086581_L11_PA), and 16 (FB_0362423_L16_PA), respectively. While for the QTLs on chromosomes 5 and 16 no significant gene involved in phenylpropene synthesis was annotated, an *isoeugenol synthase-1 like* gene (MDP0000141131) was found on chromosome 11. This gene is involved in the biosynthetic pathway of phenylpropanoid (PhP-Vs), a VOC category with multiple roles, from attractors to pollinators and defense, to important contributors of the typical ‘spicy/smoky’ aroma of fruits (Karapinar, 1990; Koeduka *et al.*, 2006; Pasay *et al.*, 2010). Isoeugenol synthase (IGS), as eugenol synthase (EGS), is an NADPH-dependent enzyme converting coniferyl acetate (synthesized from phenylanine along the phenypropanoid pathway) into isoegenol and eugenol, respectively (Koeduka *et al.*, 2009; Aragüez *et al.*, 2013). Both enzymes (IGS and EGS) can utilize the same substrate coumaryl acetate to produce *t*-anethol and chavicol, respectively. The methylation of the para-hydroxy groups on the benzene ring by O-methyltransferase (OMTs) catalyzes the final formation of *t*-anethol and estragole. The latter compound was shown to be highly accumulated in ripe fruit of ‘Royal Gala’ apple (Yauk *et al.*, 2015). In our investigation this same apple cultivar was included in a group (together with ‘Delearly’, ‘Delblush’, ‘Golden Delicious’, ‘Prima’, ‘Delicious’, and ‘Pinova’, for example) characterized by the highest accumulation of phenylpropenes during artificial chewing. The emission of PhP-Vs (phenylpropanoid volatiles) may mostly depend on the deconjugation of their glycosylate precursor, catalyzed by glycosidase upon fruit disruption, rather than its *de novo* biosynthesis (Rambla *et al.*, 2014). This mechanism, at the basis of the identification of *NSGT1* (*NON-SMOKY GLYCOSYLTRANSFERASE1*), a gene involved in the conversion of the non-cleavable triglycoside form of phenylpropanoids (preventing deglycosylation and release; Tikunov *et al.*, 2013), supports the methodology adopted here of determining VOCs during fruit artificial disruption rather than in intact fruit. The targeting of this *IGS* gene and associated SNP markers might open up more opportunities to select against or in favor of this class of compounds, important for the aromatic blend in many fruit species.

### Dynamic VOC profiling and GWAS analysis shed light on the interplay between volatilome production and fruit texture properties

**Fig. 4.**
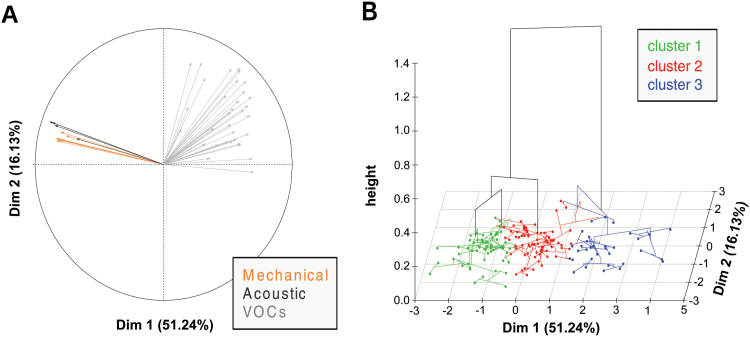
Multiple factor analysis (MFA) of texture properties (mechanical and acoustic) and VOCs. The plot in (A) shows the MFA projection of the 33 VOC masses assessed by PTR-ToF-MS at 60 s after the artificial mastication (gray color) together with eight mechanical (orange color) and four acoustic (black color) parameters. The plot in (B) depicts the hierarchical clustering of the 162 apple cultivars and their distribution over the MFA score plot defined by the first two dimensions. Apple cultivars are also grouped into three significant clusters highlighted by three colors (green, red, and blue). The detailed hierarchical clustering representation is also reported in Supplementary Fig. S4.

**Fig. 5.**
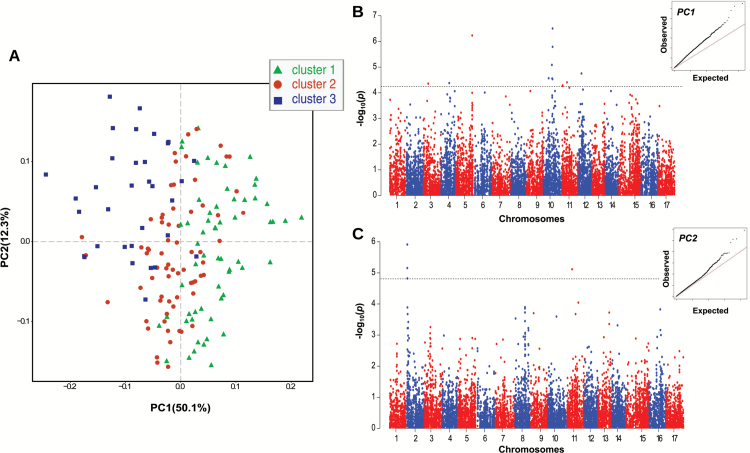
Functional principal component analysis (FPCA) of the apple volatilome assessed with PTR-ToF-MS before and after processing. In (A) the FPCA plot illustrating the distribution of the 162 apple cultivars employed here is shown. The cultivar identity is detailed in Supplementary Fig. S5. In the plot, the three clusters based on the MFA hierarchical cluster (Fig. 4) are also depicted with green triangles (Cl_1), red circles (Cl_2), and blue squares (Cl_3), respectively. In (B) and (C) the genome-wide association results for PC1 (B) and PC2 (C), respectively, are reportred. For each Manhattan plot, the Q–Q plot and the FDR correction threshold are also reported.

To examine the inter-relationship between texture properties and the aromatic pattern of apple, both the mechanic and acoustic signatures of fruit texture were assessed. The overall texture variability, examined by PCA (Supplementary Fig. S3), revealed a distinct classification of apple cultivars based on these parameters. All 162 cultivars are uniformly spread over the PCA score plot defined by the first two PCs, together expressing 97.9% of the total variability. PC1 (82.6%) corresponds to the overall apple textural performance, while PC2 (15.3%) mainly refers to the more subtle classification based on the prominence of mechanical (i.e. flesh firmness) or acoustic (i.e. crispness) parameters. Although this characterization has been performed in previous investigations (Costa *et al.*, 2011; Longhi *et al.*, 2012), the results presented here were obtained with the largest apple collection employed to date for fruit texture investigation. The texture data set was further integrated with the volatilome data (Supplementary Table S2), represented by the array of VOCs fingerprinted at 60 s after the *in vitro* mastication and assessed using an MFA (Fig. 4; Supplementary Fig. S4). In this computation, the samples were distributed according to the first two dimensions, together accounting for 67.4% of the total phenotypic variance. The loading projection (depicted in Fig. 4A) shows that VOC and texture variables were oriented oppositely, suggesting a contrasting physiological behavior. Most of the apple cultivars distinguished by high aromatic volatile production are therefore characterized by a low texture performance, and vice versa. This distinction, which is for the most part plotted according to the first dimension (explaining 51.2% of the entire variability), is moreover magnified in the hierarchical clustering represented in the MFA 2D-plot (Fig. 4B). In this plot, cultivars distributed according to their phenotypic values are also grouped into three significant clusters, defined by the specific weight of the most predominant phenotype (Supplementary Table S5; Supplementary Fig. S4). The first two clusters (cluster 1 and cluster 2) are distinguished by a low VOC production. Moreover, while in cluster 1 accessions with high acoustic performance are included, in cluster 2 apple accessions with high values for mechanical parameters are grouped. The last group, namely ‘cluster 3’, is instead represented by varieties with low texture performance but high VOC production. Although VOC and texture regulation is carried out in two distinct physiological pathways, they seem to be controlled by two mechanisms negatively correlated with each other. To shed light on this regulation, the VOC profiles were further assessed applying an FPCA (Fig. 5; Supplementary Fig. S5), which considered the entire profile of each VOC mass released during the artificial chewing process. FPCA is an exploratory multivariate technique that allows the analysis of functional data, essentially curves and trajectories. In this context, the VOCs released during the *in vitro* mastication are considered as ‘functional’, since they are single entities rather than merely sequences of individual observations (Ramsay and Silverman, 2005). The distribution of the apple accessions on the FPCA plot defined by the first two PCs (PC1, 50.1%; PC2, 12.3%) showed a consistent grouping of the varieties into three clusters (Fig. 5A) according to the previous MFA hierarchical plot (Fig. 4B).

To genetically dissect the VOC control in apple, the FPCA components were further employed as phenotypic traits in GWAS computation. The inclusion of PC1 in the analysis enabled the detection of a major QTL on chromosome 10, with a cluster of SNPs exceeding the FDR adjusted threshold (Fig. 5B; Supplementary Table S4). According to the Manhattan plot, this QTL coincided with *MdPG1*, a gene encoding a polygalacturonase involved in the depolymerization of pectin and playing a major role in the control of fruit firmness in apple (Brummell and Harpster, 2001; Brummell, 2006). The fact that this locus, known to be associated with fruit firmness variation in apple (King *et al.*, 2000; Maliepaard *et al.*, 2001; Costa *et al.*, 2010; Longhi *et al.*, 2012, 2013; Kumar *et al.*, 2013), was associated with the quantitative variation of VOC production in apple reinforces the hypothesized interplay between texture and aroma. When PC2 was instead used in the GWAS analysis, another QTL was identified and located on chromosome 2 (Fig. 5C; Supplementary Table S4). Among the SNPs exceeding the adjusted threshold, a marker associated with *MdAAT1* was found (FB_0451368_L2_PA). This gene, as reported above, is a major candidate in the formation of esters. The association between *MdAAT1* and PC2 is instead more related to the type of aroma (rather than its overall production), which in apple is for the most part related to the ratio between ester and alcohol (Newcomb *et al.*, 2006; Ulrich and Dunemann, 2012; Farneti *et al.*, 2015*a*). This result revealed that the quantitative and qualitative VOC production in apple are under different genetic control, confirmed by the different genetic association obtained when using PC1 or PC2. In particular, the association between the principal component related to the general amount of volatile and *MdPG1* supports the role of fruit texture structure in the release of aroma in apple.

### The selection in favor of fruit firmness negatively impacts the production of VOCs in apple

**Fig. 6.**
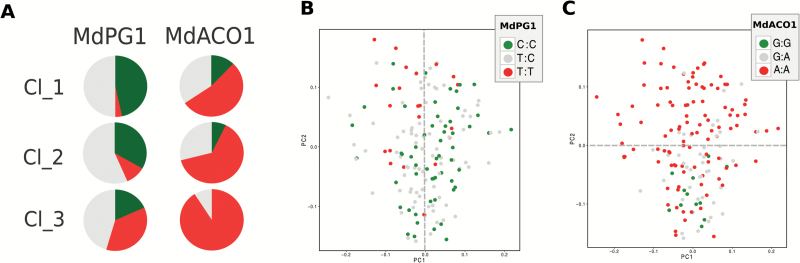
MdPG1 and MdACO1 allelotype configuration of the apple varieties distributed on the FPCA plot. For each panel, green and red color are for the favorable and unfavorable homozygous allelic state, while the heterozygous allelic state is reported in gray. The pie charts depicted in (A) show the proportion of the allele for the two functional markers in the three clusters defined by the MFA analysis. (B) and (C) The cultivar distribution of the FPCA plot, colored according to the allelotype of MdPG1 (B) and MdACO1 (C), respectively. In the key of (B) and (C), the allelism of the two SNPs associated with both genes is also indicated.

Within the several aspects of apple fruit quality, fruit texture and flavor are dominant features for their effect on the post-harvest performance and consumer preference (Baietto and Wilson, 2015). Despite the relevance of both traits, the breeding for fruit quality is fundamentally based on fruit firmness, for two reasons. Fruit texture (especially firmness) is easy to measure, and validated functional markers are already available for an anticipated assisted selection of the best performing individuals, such as MdACS1, MdACO1, and MdPG1 (Costa *et al.*, 2005; Zhu and Barritt, 2008; Baumgartner *et al.*, 2016). Also for fruit aroma a molecular marker based on the *alcohol acyltransferase* gene has recently been developed (MdAAT1; Dunemann *et al.*, 2012). However, since an association between the type of aroma selected by this marker and consumer preference is lacking, this tool is not yet used in breeding-assisted selection. To this end, for a better understanding of the relationship between fruit texture and aroma, the distribution of the apple accessions over the FPCA and arranged into three clusters (Fig. 5A; Supplementary Table S5) was reconsidered on the basis of the allelotype configuration of two functional markers, MdPG1 and MdACO1 (Fig. 6A). Amongst others, these two markers were specifically selected for sharing their genetic position on chromosome 10, co-locating with QTLs associated with fruit firmness and softening (Costa *et al.*, 2010; Longhi *et al.*, 2012; Kumar *et al.*, 2013). The distribution of the apple cultivars based on MdPG1 allelism showed a clear distinction around PC1 (Fig. 6B). While most of the cultivars characterized by a homozygous unfavorable allelic configuration (TT), promoting fruit softening due to an intense pectin enzymatic depolymerization, are plotted on the negative PC1 area, the favorable homozygous genotypes (CC) are mostly located on the positive PC1 quadrants. This distribution confirmed, moreover, the distinction of the three clusters identified through both MFA and FPCA (Figs 4, 5). According to this new elaboration, cluster 3 (represented by varieties with low texture properties and high VOC production) was distinguished by a high proportion of unfavorable TT alleles for MdPG1, with regards to clusters 1 and 2. The allelic distribution for MdPG1 is moreover consistent with that of MdACO1. Cluster 3 is therefore characterized by the dominant presence of the AA allelotype for this gene, associated with a high ethylene production, while in cluster 1 and 2 this ratio decreases in favor of the GG allelotype, which is associated with a low ethylene production (Fig. 6A). The varieties distinguished by the two MdACO1 allelotypes are furthermore distributed around PC2 (Fig. 6C), with the cultivars included in cluster 3 mostly plotted in the PC2 positive area. The breeding in favor of firm fruit would therefore select apple fruit also distinguished by a low aroma production. This relationship can be explained in relation to the physiological role of the two genes employed here. *MdPG1* is involved in the control of fruit texture, which depends on the degradation of the cell wall polysaccharide structure. Firm apples are therefore characterized by a more intact and solid cell wall, which can prevent the release of VOCs due to tighter cell compartmentation. On the other hand, *MdACO1* is the last gene governing the final biochemical formation of ethylene, a plant hormone triggering and co-ordinating several ripening processes (Giovannoni, 2001; Bennett and Labavitch, 2008). The amount of ethylene, moreover, has already been correlated with fruit softening in apple (Costa *et al.*, 2005, 2010; Wakasa *et al.*, 2006) as well as the rate of VOC production (Schaffer *et al.*, 2007). To this end, the actual breeding in favor of post-harvest would most probably also decrease the aromatic blend in apple fruit.

The exploitation of the genetic variability existing within the apple germplasm can allow a valuable combination of alleles for the selection of a high quality ideotype. This goal, however, can be achieved only with a more informed and precise identification of the best performing cultivars to be used as superior parental lines.

## Supplementary Material

Supplementary Data
