# Supplementary material for: Genome-wide association study unravels the genetic control of the apple volatilome and its interplay with fruit texture
Source: J Exp Bot. 2017 Feb 24;68(7):1467–78. doi: 10.1093/jxb/erx018 (PMC5441895; doi:10.1093/jxb/erx018)
Supplement: Supplementary Data [file erx018_Supplementary_Data.zip › supplementary_figures_S1_S5_tables_S1_S3_S5.pdf]

Supplementary Data

Genome-wide association study unravels the genetic control of the apple volatilome and its interplay with fruit texture

Brian Farneti, Mario Di Guardo, Iuliia Khomenko, Luca Cappellin, Franco Biasioli, Riccardo Velasco, Fabrizio Costa*

*Corresponding author:
Fabrizio Costa
Research and Innovation Centre, Fondazione Edmund Mach
Via Mach 1, 38010 San Michele all'Adige, Trento, Italy
Phone: 0039-0461-615358
E-mail: fabrizio.costa@fmach.it

Keywords: Volatilome, VOCs, PTR-ToF-MS, Fruit texture, GWAS, SNP, Ester, Phenylpropene, multi-factorial analysis, functional principal component analysis.

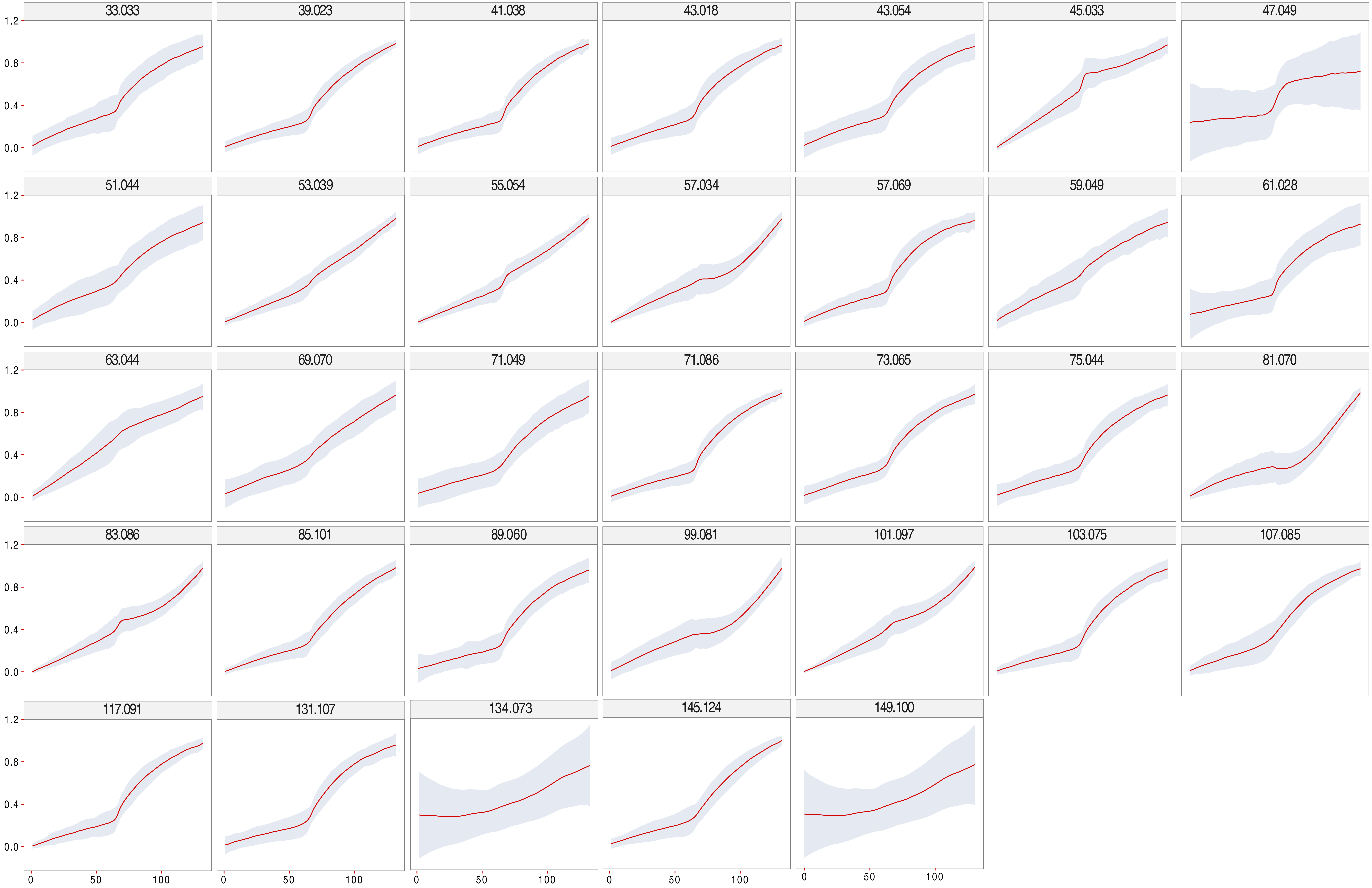

Figure S1. VOC dynamics assessed by PTR-ToF-MS coupled with the artificial chewing device. Each graph shows the normalized VOC concentration (scaled between 0 and 1) of each mass plus the standard deviation calculated over the 162 cv.

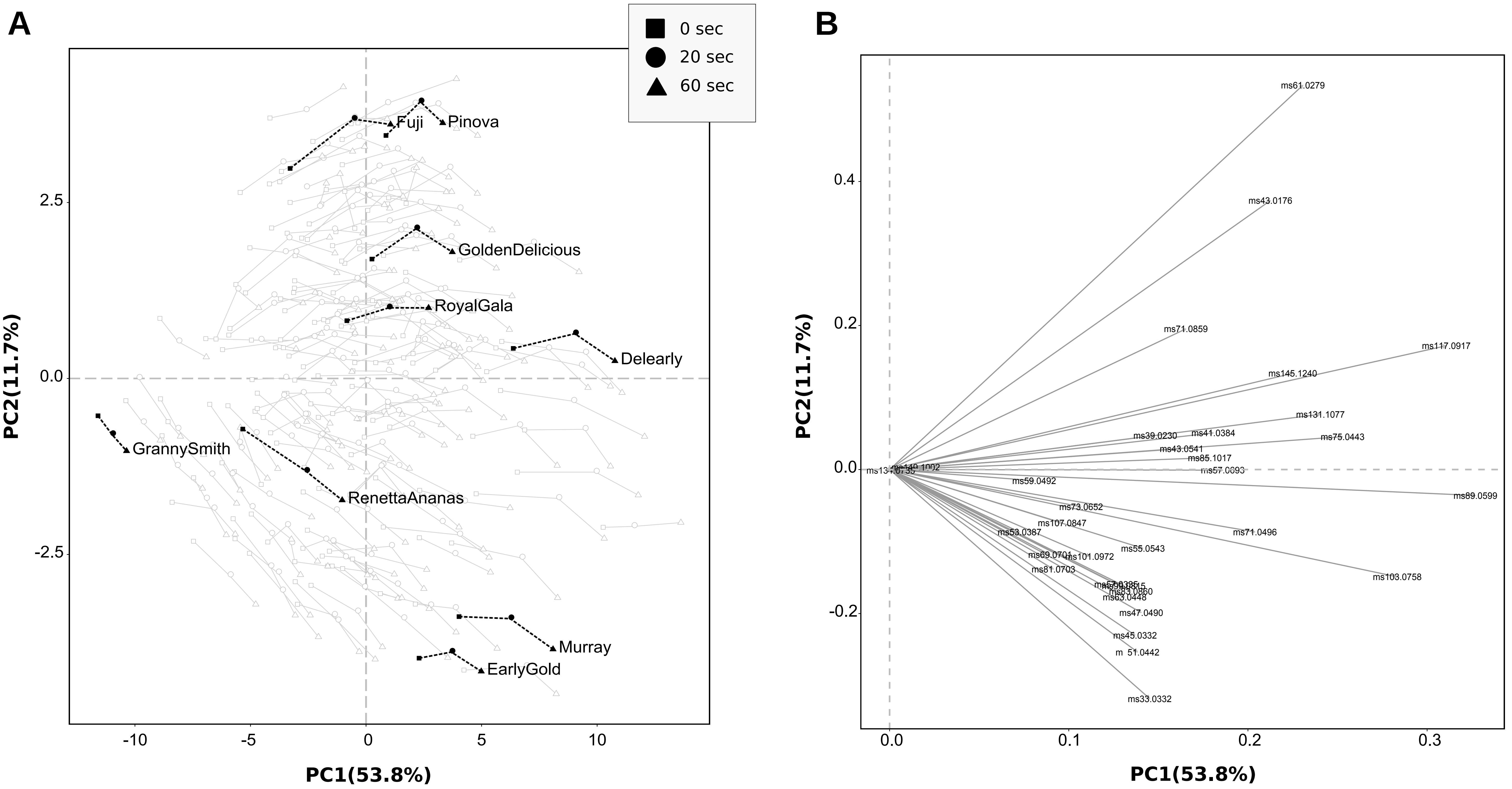

Figure S2. High resolution vectorial image of the Principle Component Analysis (PCA) plot (A) and loading projection (B) illustrated in Fig. 2.

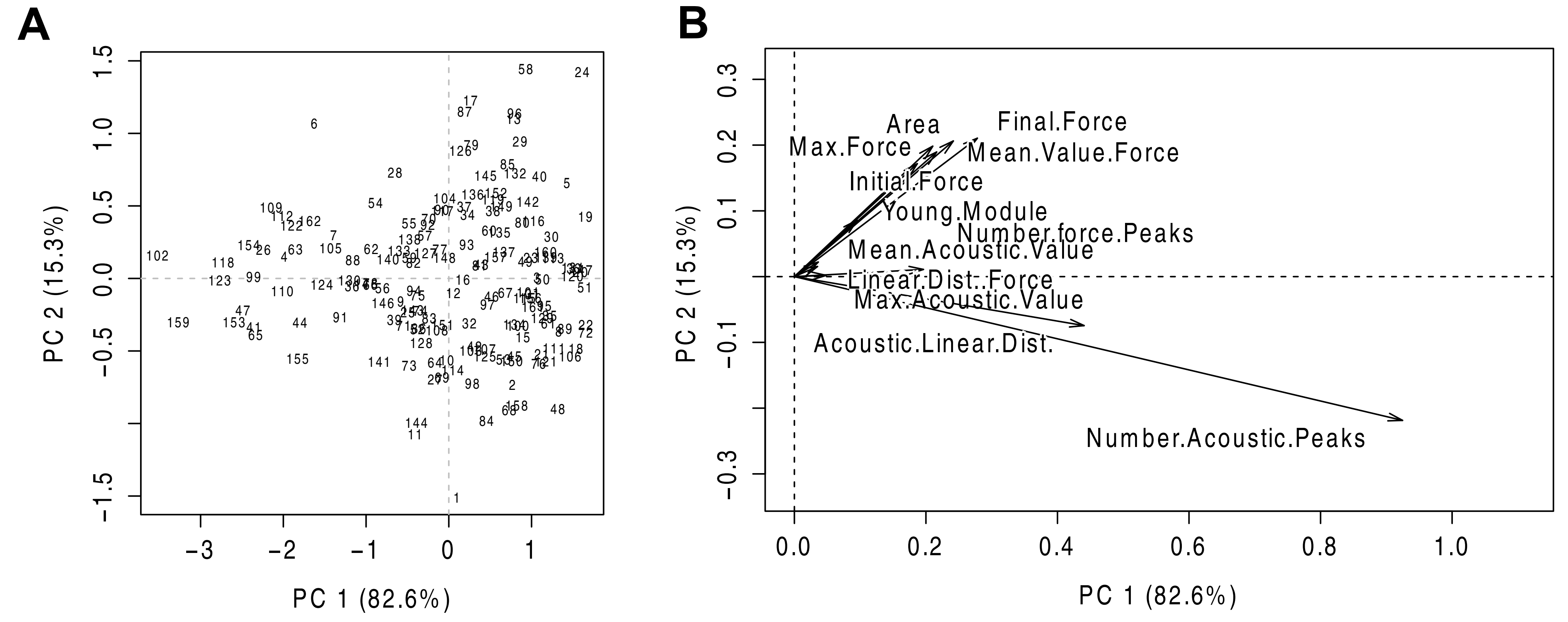

Figure S3. Principle Component Analysis (PCA) plot (A) and loading projection (B) of texture parameters. .

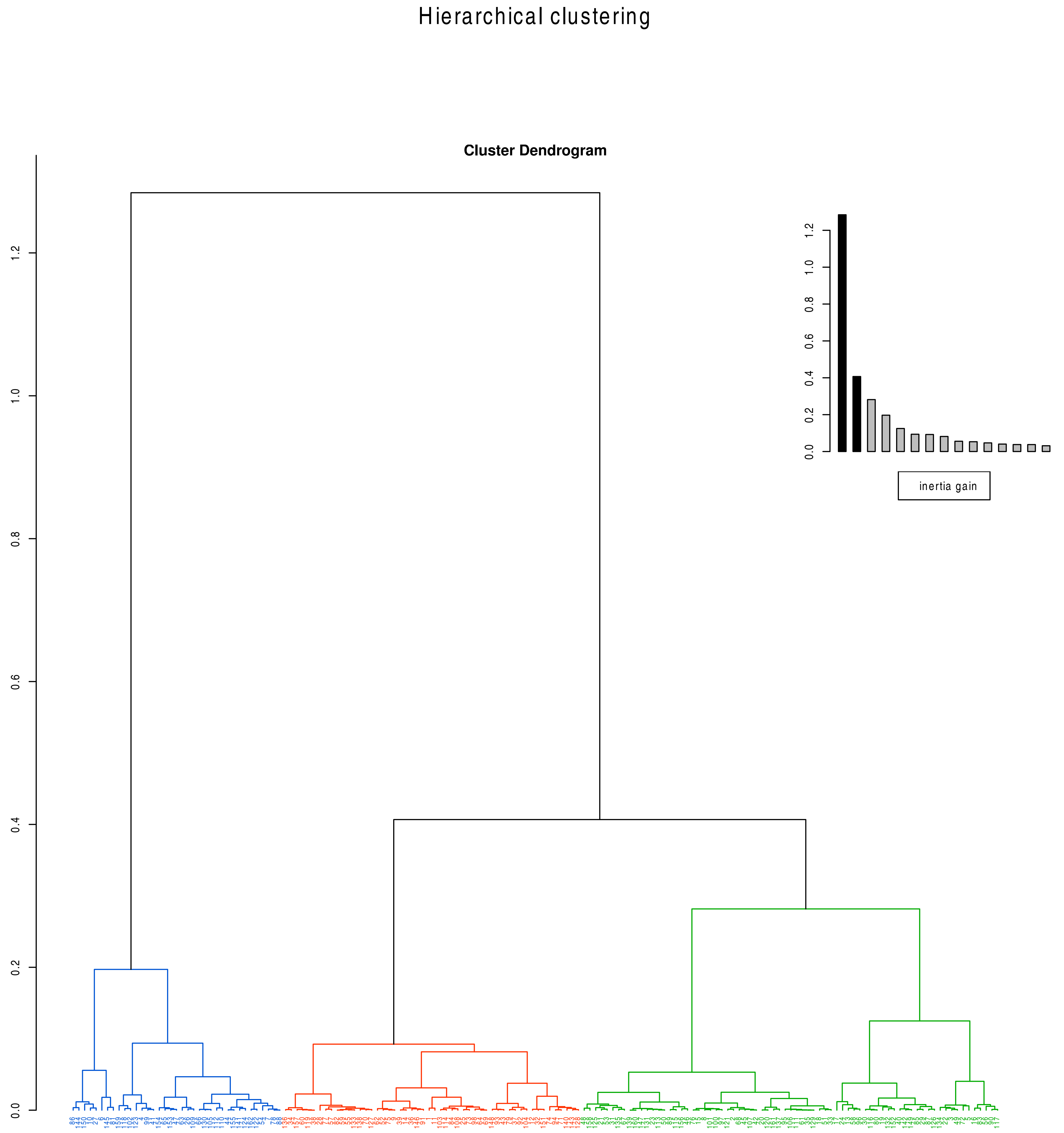

Figure S4. High resolution vectorial immage of the MFA hierarchical clustering. Significant clusters are highlighted in green (Cl_1), orange (Cl_2) and green (Cl_3).

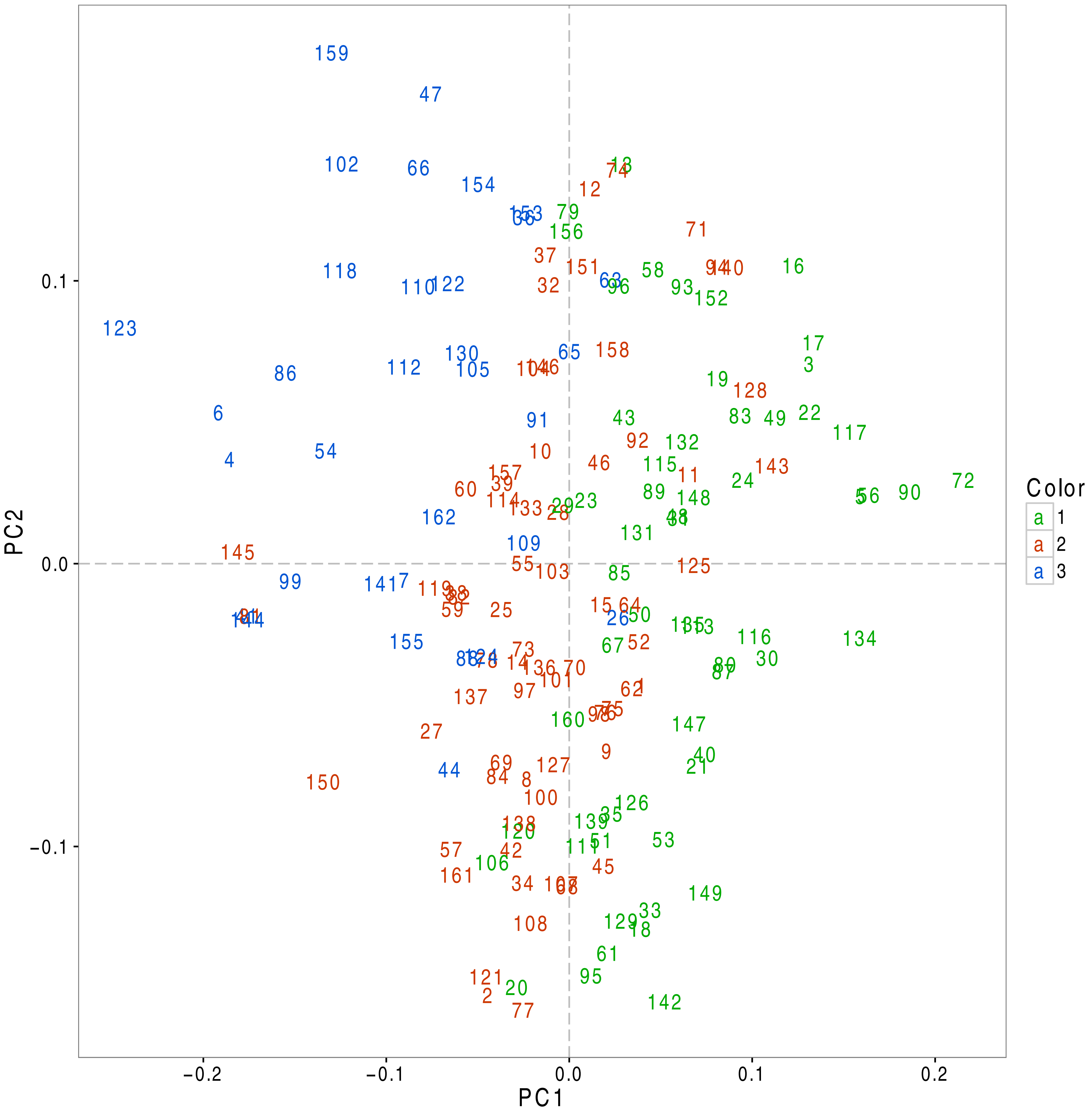

Figure S5. High resolution vectorial image of the Functional Principle Component Analysis (FPCA) plot of VOCs illustrated in Fig. 5. Varieties are coded with numbers, specified in Supplementary Tab. S3.

Table S1. List of mechanical and acoustic texture parameters.

Parameter	***Descript on***	***Unit***
Mechanical parameters		
Yield force	Force measured at the yield point (init al phase)	N
Maximum force	Maximum force value recorded over the probe's travel	N
Final force	Force measured at the end of the probe's travel	N
Mean force	Mean force value over the ent re mechanical prof le	N
Area	Area underlying the mechanical prof le	N%
Force linear distance	Computat on of the force curve length	-
Young's module	Elast city module, computed as rat o between stress and strain	N%
N. Force peak	Number of counted force peaks	-
Acoust c parameters		
N. Acoust c peak	Number of the acoust c peaks calculated above the threshold of 10 dB	-
Maximum acoust c pressure	Highest acoust c peaks detected on the sound pressure wave	dB
Mean acoust c pressure	Mean value of the sound pressure recorded over the acoust c prof le	dB
Acoust c linear distance	Computed length of the acoust c prof le	-

Table S3. Volatile compounds detected by PTR-ToF-MS. In the table the number referring to the CAN groups are also reported.

m/z	Group	Formula	ID	Annotation
**33.033**	5	CH5O+	Alcohol	Methanol
**39.023**	3	C3H3+	General fragment	General fragment
**41.038**	3	C3H5+	General fragment	Alcohols and esters
**43.018**	1	C2H3O+	Ester fragment	Ester fragment
**43.054**	3	C3H7+	Alcohol	Propanol, general fragment
**45.033**	1	C2H5O+	Carbonyl compound	Acetaldehyde
**47.049**	1	C2H7O2+	Alcohol	Ethanol
**51.044**	5	CH3OH*H3O+	Alcohol	Methanol cluster
**53.039**	7	C4H5+	n.a.	n.a.
**55.054**	4	C4H7+	n.a.	n.a.
**57.034**	4	C3H5O+	General fragment	Aldehydes (Trans-2-hexenal, Hexanal, Decanal), esters (Methyl 2-methyl butanoate, Ethyl 2-methyl butanoate, Butyl propionate, Hexyl acetate, Propyl propanoate)
**57.069**	3	C4H9+	Alcohol	Alcohols (1-Butanol, 1-Pentanol, 1-Hexanol,2-Methyl-1-propanol, Pentanol, Iso-pentanol, 1-Heptanol, 2-Ethyl-1-hexanol, Octanol, Nonanol)
**59.049**		C3H7O+	Carbonyl compound	Acetone
**61.028**	1	C2H5O2+	Ester	Esters (Ethyl acetate, Butyl acetate, 2-Methylbutyl acetate, Hexyl acetate, propyl acetate, isobutyl acetate, Isoamyl acetate, Amyl acetate), Acetic acid
**63.044**	2	C2H7O2+	Alcohol	Ethylene glycol
**69.070**	7	C5H9+	Carbonyl compound	Sesquiterpenes fragment (alpha-Farnesene), other VOCs
**71.049**	2	C4H7O+	Ester	Esters (Ethyl butanoate, Ethyl hexanoate, Propyl butanoate, Butyl butanoate, 2-Methylbutyl acetate, Isoamyl acetate)
**71.086**		C5H11+	Alcohol	Alcohols (2-Methyl-butanol, 3-Methyl-butanol, Pentanol, Iso-pentanol, 2-ethyl-1-hexanol, Octanol, Nonanol)
**73.065**	1	C4H9O+	Carbonyl compound	Butanal
**75.044**	1	C3H7O2+	Ester	Butyl propanoate
**81.070**	4	C6H9+	Carbonyl compound	Fragment of Terpenes (Linalool), sesquiterpenes (alpha-Farnesene), aldehydes (trans-2-hexenal)
**83.086**	4	C6H11+	General fragment	Alcohols ((Z)-3-Hexen-1-ol, (E)-2-Hexen-1-ol, 5-Hexen-1-ol), Aldehydes (Hexanal), Sesquiterpenes (alpha-Farnesene)
**85.101**	3	C6H13+	Alcohol	Alcohols (1-Hexanol, Nonanol)
**89.060**	1	C4H9O2+	Ester	Esters (Ethyl acetate, Ethyl butanoate, Propyl butanoate, Butyl butanoate)
**99.081**	4	C6H11O+	General fragment	Aldehydes (trans-2-hexenal), esters (Ethyl hexanoate, Hexyl acetate)
**101.097**	4	C6H13O+	Carbonyl compound	Aldehydes (Hexanal)
**103.075**	2	C5H11O2+	Ester	Esters (Methyl butanoate, Ethyl 2-methyl butanoate, propyl acetate)
**107.085**	2	C8H11+	n.a.	n.a.
**117.091**	2	C6H13O2+	Ester	Esters (Methyl 2-methyl butanoate, Ethyl butanoate, Ethyl hexanoate, Butyl acetate, Isobutyl acetate, Propyl propanoate, Amyl acetate)
**131.107**	2	C7H15O2+	Ester	Esters (Ethyl 2-methyl butanoate, Butyl propionate, 2-Methylbutyl acetate, Isoamyl acetate, Propyl butanoate, Amyl acetate)
**134.073**	6		Phenyl fragment	Phenyl fragment (Estragole, Anethol)
**145.124**	2	C8H17O2+	Ester	Esters (Ethyl hexanoate, Butyl butanoate)
**149.096**	6	C10H13O+	Phenylpropenes	Phenylpropenes (Estragole, Anethol)

Table S5. List of apple cultivars employed in this study. Accessions are divided into 3 groups based on the MFA hierarchical clustering. For each cluster, N° is the numerical code as reported in Supplementary Fig. S3, Fig. S4 and Fig. S5, while cultivar is the name of the apple accession included in the germplasm collection used in this survey.

Cluster 1

N°	*Cultivar*
3	Angold
5	Annurca
13	Ben Devis
16	Cakanska Pozna
17	Calamari
18	Cameo
19	Carola
20	Catarina
21	Cauflight
22	Challenger
23	Clivia
24	Commercio
29	Coop 28
30	Coop 30
31	Coop 36
33	Coop 8
35	Crimson Snow
40	Delcoros
43	Delprim
48	Early Red Stayman
49	Edel Bohmer
50	EdenSpur
51	Elite
53	Emilia B9
56	Fiamma
58	Fragoni
61	Fuji
67	Gloster
72	Granny Smith
79	James Grieve
80	Jeromine
83	Jonagold
85	Jonica
87	Kinsey
89	Lygol
90	Limoncini
93	Magnolia Gold
95	Mairac
96	Mela D'inverno
106	Nicogreen
111	Okanoma
113	Orin
115	Ozark Gold
116	Paragon Winesap
117	Permain Dorato
120	Pink Rose
126	Primiera
129	Red Chief
131	Reggent
132	Renetta Ananas
134	Rosa Di Caldaro
135	Rosmarina Rossa
139	Ruby
142	Santared Fireside
147	Smeralda
148	Smith Jonathan
149	Sonya
152	Striato Dolce
156	Surprise
160	Turner Delicious

Cluster 2

N°	*Cultivar*
1	Abbondanza
2	Almagold
8	Auvil Spur
9	Badami Golden
10	Beauty Of Bath
11	Belfiore Giallo
12	Bella Di Boskoop
14	Blairmont
15	Boutabguia
25	Coop 10
27	Coop 16
28	Coop 26
32	Coop 7
34	Cornell Red
37	Damziger Kanapfel
38	Del Blush
39	Delbard Estivale
42	Delicious
45	Diwa
46	Durezza Verde
52	Elstar
55	Festival
57	Florina
59	Freedom
60	Freiburg
62	Gaia
64	Gallia Beauty
68	Gold Chief
69	Golden Delicious
70	Goldjon
71	Goro
73	Greendale
74	Greensleves
75	Grimes Golden
76	Holiday
77	Idagold
78	Ingrid Marie
81	Jolly
82	Jonadel
84	Jonasty
92	Locale Pergine
94	Magrè
97	Mela Forestiera
98	Melrose
100	Morspur Red
101	Mosebar
103	Mutsu
104	Nabella
107	Norten Spy
108	Nugget
114	Orleans
119	Pink Pell
121	Pinova
125	Primgold
127	Reanda
128	Red Baron
133	Renoire
136	Royal Gala
137	Royal Red Delicious
138	Rubicon
140	San Lugano
143	Saturn
145	Seriana
146	Shenandoah
150	Spartan
151	Spokane Beauty
157	Tavola Bianca
158	Topaz
161	Wellspur Delicious

Cluster 3

N°	*Cultivar*
4	Anna
6	Apollo
7	Aranciata
26	Coop 15
36	Croncels
41	Delearly
44	Delshel
47	Early Gold
54	Empress
63	Galbered Apfel
65	Geneva
66	Ginger Gold
86	Kendal
88	Lampone
91	Lired
99	Merrigold
102	Murray
105	Niagara
109	Odin
110	Okanagan
112	Oldemburg x Cox
118	Petrel
122	Piros
123	Precoce Rigotti
124	Prima
130	Redfree
141	Sansa
144	Selena
153	Summer Champion
154	Summer Red
155	Sunrise
159	Tunda
162	Worcester Permain
